# Human African Trypanosomiasis Research Gets a Boost: Unraveling the Tsetse Genome

**DOI:** 10.1371/journal.pntd.0002624

**Published:** 2014-04-24

**Authors:** Serap Aksoy, Geoffrey Attardo, Matt Berriman, Alan Christoffels, Mike Lehane, Dan Masiga, Yeya Toure

**Affiliations:** 1 Yale School of Public Health, Department of Epidemiology and Public Health, New Haven, Connecticut, United States of America; 2 The Wellcome Trust Sanger Institute, Wellcome Trust Genome Campus Hinxton, United Kingdom; 3 South African National Bioinformatics Institute, MRC Bioinformatics Unit, University of the Western Cape, Bellville, South Africa; 4 Vector Group, Liverpool School of Tropical Medicine, Pembroke Place, Liverpool, United Kingdom; 5 Molecular Biology and Bioinformatics Unit, International Centre of Insect Physiology and Ecology (icipe), Nairobi, Kenya; 6 Vector, Environment and Society Unit, Tropical Diseases Research (TDR), World Health Organization, Geneva, Switzerland

Human African trypanosomiasis (HAT), also known as sleeping sickness, is a neglected disease that impacts 70 million people distributed over 1.55 million km^2^ in sub-Saharan Africa [1]. *Trypanosoma brucei gambiense* accounts for almost 90% of the infections in central and western Africa, the remaining infections being from *T. b. rhodesiense* in eastern Africa [1]. Furthermore, the animal diseases caused by related parasites inflict major economic losses to countries already strained [2]. The parasites are transmitted to the mammalian hosts through the bite of an infected tsetse fly.

In the early part of the 20th century, HAT epidemics decimated human populations in many parts of Africa. In the 1930s, systematic screening, treatment, and follow-up of millions of individuals by the colonial administrations led to a dramatic decrease in disease transmission (reviewed in [3]). Following independence in the 1950–60s, HAT control efforts in most African countries were relaxed and taken over by other priorities in light of the decline in disease incidence. Unfortunately, the disease slowly returned, with flare-ups beginning to be reported throughout the endemic areas by the late 80s and early 90s [4], [5], [6]. In a 1997 resolution, WHO strongly advocated access to diagnosis and treatment with surveillance and control activities, concurrently setting up a network to strengthen coordination among endemic countries [7], [8]. Sadly, the disease killed thousands of people before control measures began to take effect [9]. In 2006, 20 out of the 36 endemic countries had achieved the target of no new cases, and eight countries reported fewer than 100 cases. In 2008, WHO declared that the newly reported cases had dramatically declined to fewer than 10,000 continent-wide and called for plans towards a HAT elimination policy [10]. The *gambiense* form of the disease has been targeted for elimination by 2020 [11]. It is important to note, however, that estimating the true burden of HAT is difficult, as the disease affects the most neglected populations, living in remote and rural settings where the majority of people affected are beyond the reach of health care systems and are not reported in the health metrics [12]. It is also important to be mindful of ongoing political conflicts, which stand to refuel the emergence of epidemics unless control measures continue to be employed in endemic countries [13], [14], [15]. Thus, it is imperative that endemic countries must at least continue to retain mechanisms and health personnel who can recognize and report potential HAT cases to prevent reemergence of the disease [16]. A flexible set of control efforts needs to be adapted to the different epidemiological patterns in order to adopt the most adequate strategies for maintaining cost-effectiveness [17], [18].

Achieving disease control in the mammalian host has been challenging given the lack of effective mammalian vaccines and cheap and easily deliverable drugs. Furthermore, at times of low endemicity, relying on active surveillance of human infections is not cost-effective. The challenge now is to identify control methods that will ensure that the continent remains free of HAT. One approach that has worked well to curb disease is the reduction of tsetse vector populations. This is due to the low reproductive rate of tsetse resulting from its viviparous biology. Improving the efficacy of the currently available vector control tools (targets, traps, and insecticide applications) or enhancing the implementation of control programs that utilize this set of tools in different ecological settings can improve effectiveness. Also, the effectiveness of these tools can be improved through a better understanding of tsetse physiology—a feature that has enabled the development of these tools in the first place. The complete genome sequence information from *Glossina morsitans morsitans*, published in *Science* this week, now provides a unique opportunity to transform tsetse research and disease control practices [19]. Particularly important in this regard is knowledge of tsetse's vision, olfactory, immune, digestive and reproductive physiology. The eight research papers published this week PLOS-wide that accompany the tsetse genome paper already point to unique opportunities for improving control (see Tsetse Genome Biology Collection).

Getting at the genome data, however, has not been an easy road. The *Glossina* community was small, and many of the facilities in Africa that maintained tsetse fly colonies and conducted research on this vector were beginning to downsize their programs in the early 2000s due to reduced research funds, despite the rising disease incidence. A small group of researchers, however, argued that moving tsetse research into the -omics era would provide new stimulus that would give rise to opportunities for control and, importantly, would attract young researchers to the field of tsetse research [20]. The Molecular Entomology work area of the Special Programme for Research and Training in Tropical Diseases (TDR) at the World Health Organization (WHO) funded the establishment of a consortium (International *Glossina* Genome Initiative [IGGI]) in 2004 that brought together an interdisciplinary group of researchers from multiple institutions to chart the course towards the finish line [20], [21]. The consortium recruited global funds that enabled the development of a molecular toolbox, which initially included data from several large expressed-sequence (EST) libraries along with construction and sequencing of a Bacterial Artificial Chromosome (BAC) library. Given that the tsetse vector and the disease is endemic to sub-Sahara Africa, IGGI membership chose to use this networking opportunity to help build research capacity in Africa on genetics and genomics aspects of tsetse [21]. In the following seven years, the IGGI consortium met yearly for coordination meetings, organized five bioinformatics workshops that trained students and junior faculty from endemic countries, and set up exchange programs for students and researchers from Africa to be trained in research laboratories in Europe and the United States. Over 40 African students and researchers from African institutions attended a transcriptome analysis jamboree held at the South African National Bioinformatics Institute (SANBI) in 2007. The genome project led by the Sanger Institute also benefited from the contributions of many genome centers, including TIGR in the US, RIKEN in Japan, and Genoscope in France. Two manual community annotation workshops and the 146 experts recruited from the broad vector community helped the program to reach the finish line this month. Building on their success story with *Glossina morsitans* genome, the consortium has now secured funds from the National Institute of Health in the US to sequence five additional species of *Glossina* at the University of Washington Genome Center, and this project is nearly complete.

The PLOS-wide collection, titled Tsetse Genome Biology, accompanying this issue presents two Historical Perspective articles that review events related to the devastating HAT epidemics in the early years of the 20th century [22], [23]. In addition, the collection has eight research articles that expand on the genome discoveries and report on several low hanging fruits that are ready for exploitation for improved vector control. One of these discoveries is related to the tsetse olfactory system, which appears to be significantly streamlined when compared to other disease vectors, such as mosquitoes [24]. At the core of the success of the trapping devices is the ability of tsetse to recognize the color blue and be attracted to certain smells, which are used as bait. The availability of the full spectrum of olfactory components now stands to provide new or more effective species-specific attractants that can improve the efficacy of traps. Another area that is unique among disease vectors is tsetse's viviparous reproductive system, which involves the production of one progeny at a time that is nourished by the milk secretions of the mother during intrauterine development. Each female can produce on average eight to ten progeny, and she remains fecund during much of her adult life. This low reproductive capacity is at the core of the success of strategies that aim to reduce tsetse populations. To nurture its developing larva, the tsetse female lactates and produces milk. Researchers have now identified a previously unknown group of indispensable milk proteins that are coordinately synthesized during the lactation phase. Without these proteins, the female cannot support her developing larva [25]. Interestingly, a single transcription factor, the homeobox factor Ladybird Late, may be responsible for the coordinated expression of all milk proteins, opening the way for future novel biological strategies that target tsetse's lactation cycle and associated reproductive capacity [26]. In addition, lactation-specific aquaporin proteins were also identified and are needed for water transport and hydration during milk synthesis [27]. Finally, comparison of before and after pregnancy transcriptomes indicates that prevention of oxidative stress may be the key for the success of the prolonged reproductive output and longevity associated with tsetse female physiology [28].

Tsetse carries with it multiple symbiotic microbes, one of which is called *Wolbachia. Wolbachia* has been shown to manipulate host reproductive physiology in many insects, including in tsetse [29]. One of the research papers in the collection describes the Whole Genome Sequence of the *Wolbachia* symbiont obtained from the same *Glossina morsitans* species [30]. This study also reports that unusually large sections of the genome of the *Wolbachia* symbiont have been transferred to the host tsetse genome, particularly residing in the sex chromosomes [30].

Finally, although extensive research has been conducted on African trypanosomes in the mammalian host, knowledge of tsetse–parasite interactions remains sparse. An area of interest has been discovery of tsetse mechanisms that can block parasite transmission either in the midgut or in the salivary glands. This is of interest to both basic and applied research since the ability to engineer greater resistance in flies could solve the problem of disease transmission. Researchers characterized important tsetse genes whose products are components of physical and immunological barriers in the gut Peritrophic Matrix (PM) to trypanosome infections [31]. Another study has compared trypanosome and tsetse transcriptomes from normal and parasitized salivary glands, as well as from normal and parasitized mammalian blood [32]. This study reports on parasite adaptations that may enable its survival in the two different host environments and host gene expression modifications that may help parasite survival in either the salivary gland or, possibly, in the mammalian bite site [32].

The wealth of information that is revealed from the genome and functional genomics data is now ready to be explored and exploited. The soon-to-be-available additional *Glossina* genomes will shed light onto the species-specific habitat and vertebrate host requirements and varying vector competence associated with the different species. Thus, money was well spent on the small investment made by WHO-TDR to bring together IGGI. An important goal of the genome project was expansion of the community of tsetse researchers—the consortium now invites all interested researchers, particularly junior scientists, to take a look at research opportunities on tsetse and trypanosomes. The consortium hopes that while HAT is a neglected tropical disease, vector tsetse research may enjoy broader participation from the vector community and lead to improved and/or novel methods to eliminate disease.

## References

[1] Simarro PP, Cecchi G, Franco JR, Paone M, Diarra A, et al (2012) Estimating and mapping the population at risk of sleeping sickness. PLoS Negl Trop Dis 6: e1859. 23145192 10.1371/journal.pntd.0001859 PMC3493382

[2] Shaw AP (2004) Economics of African Trypanosomiasis. In: Maudlin I, Holmes PH, Miles MA, editors. The Trypanosomiases. Wallingford: CABI Publishing. pp. 369–402.

[3] Cox FE (2004) History of sleeping sickness (African trypanosomiasis). Infect Dis Clin North Am 18: 231–245. 15145378 10.1016/j.idc.2004.01.004

[4] van Hove D (1997) Sleeping sickness in Zaire. Lancet 349: 438. 10.1016/S0140-6736(97)80068-2 9033508

[5] Moore A, Richer M, Enrile M, Losio E, Roberts J, et al (1999) Resurgence of sleeping sickness in Tambura County, Sudan. Am J Trop Med Hyg 61: 315–318. 10463686 10.4269/ajtmh.1999.61.315

[6] Ekwanzala M, Pepin J, Khonde N, Molisho S, Bruneel H, et al (1996) In the heart of darkness: sleeping sickness in Zaire. Lancet 348: 1427–1430. 8937285 10.1016/S0140-6736(96)06088-6

[7] WHO (1997) Resolution 50.36, 50th World Health Assembly. Geneva: World Health Organization.

[8] Simarro PP, Louis FJ, Jannin J (2003) Sleeping sickness, forgotten illness: what are the consequences in the field? Med Trop (Mars) 63: 231–235. 14579457

[9] Simarro PP, Diarra A, Ruiz Postigo JA, Franco JR, Jannin JG (2011) The human African trypanosomiasis control and surveillance programme of the World Health Organization 2000–2009: the way forward. PLoS Negl Trop Dis 5: e1007. 21364972 10.1371/journal.pntd.0001007 PMC3042999

[10] Simarro PP, Jannin J, Cattand P (2008) Eliminating human African trypanosomiasis: where do we stand and what comes next? PLoS Med 5: e55. 18303943 10.1371/journal.pmed.0050055 PMC2253612

[11] BaMGFP (2013) Private and Public Partners Unite to Combat 10 Neglected Tropical Diseases by 2020. Available: http://www.gatesfoundation.org/media-center/press-releases/2012/01/private-and-public-partners-unite-to-combat-10-neglected-tropical-diseases-by-2020. Accessed 7 April 2014.

[12] Matemba LE, Fevre EM, Kibona SN, Picozzi K, Cleaveland S, et al (2010) Quantifying the burden of rhodesiense sleeping sickness in Urambo District, Tanzania. PLoS Negl Trop Dis 4: e868. 21072230 10.1371/journal.pntd.0000868 PMC2970539

[13] Ruiz-Postigo JA, Franco JR, Lado M, Simarro PP (2012) Human African trypanosomiasis in South Sudan: how can we prevent a new epidemic? PLoS Negl Trop Dis 6: e1541. 22666506 10.1371/journal.pntd.0001541 PMC3362634

[14] Mumba D, Bohorquez E, Messina J, Kande V, Taylor SM, et al (2011) Prevalence of human African trypanosomiasis in the Democratic Republic of the Congo. PLoS Negl Trop Dis 5: e1246. 21829736 10.1371/journal.pntd.0001246 PMC3149009

[15] Rimoin AW, Hotez PJ (2013) NTDs in the Heart of Darkness: The Democratic Republic of Congo's Unknown Burden of Neglected Tropical Diseases. PLoS Negl Trop Dis 7: e2118. 23936557 10.1371/journal.pntd.0002118 PMC3723541

[16] Aksoy S (2011) Sleeping sickness elimination in sight: time to celebrate and reflect, but not relax. PLoS Negl Trop Dis 5: e1008. 21364971 10.1371/journal.pntd.0001008 PMC3042998

[17] Simarro PP, Franco JR, Diarra A, Ruiz Postigo JA, Jannin J (2013) Diversity of human African trypanosomiasis epidemiological settings requires fine-tuning control strategies to facilitate disease elimination. Res Rep Trop Med 1–6. 10.2147/RRTM.S40157 PMC6067614 30100778

[18] Solano P, Torr SJ, Lehane MJ (2013) Is vector control needed to eliminate gambiense human African trypanosomiasis? Front Cell Infect Microbiol 3: 33. 23914350 10.3389/fcimb.2013.00033 PMC3728477

[19] International *Glossina* Genome Initiative (2014) Genome Sequence of the Tsetse Fly (*Glossina morsitans*): Vector of African Trypanosomiasis. Science 344 doi:10.1126/science.1249656 10.1126/science.1249656 PMC4077534 24763584

[20] Aksoy S, Berriman M, Hall N, Hattori M, Hide W, et al (2005) A case for a *Glossina* genome project. Trends Parasitol 21: 107–111. 15734656 10.1016/j.pt.2005.01.006

[21] Butler D (2004) African labs win major role in tsetse-fly genome project. Nature 427: 384. 10.1038/427384b 14749791

[22] Headrick D (2014) Sleeping Sickness Epidemics and Colonial Responses in East and Central Africa, 1900–1940. PLoS Negl Trop Dis 8: e2772 doi:10.1371/journal.pntd.0002772 24763309 10.1371/journal.pntd.0002772 PMC3998934

[23] Mbopi-Keou FX, Belec L, Milleliri J-M, Teo C-G (2014) The Legacies of Eugene Jamot and La Jamotique. PLoS Negl Trop Dis 8: e2635 doi:10.1371/journal.pntd.0002635 24762970 10.1371/journal.pntd.0002635 PMC3998925

[24] Obiero GFO, Nyanjom SRG, Mireji PO, Christoffels A, Robertson HM, et al (2014) Odorant and gustatory receptors in the tsetse fly *Glossina morsitans morsitans* . PLoS Negl Trop Dis 8: e2663 doi:10.1371/journal.pntd.0002663 24763191 10.1371/journal.pntd.0002663 PMC3998910

[25] Benoit JB, Attardo GM, Michalkova V, Krause TB, Bohova J, et al (2014) A novel highly divergent protein family from a viviparous insect identified by RNA-seq analysis: a potential target for tsetse fly-specific abortifacients. PLoS Genet 10: e1003874 doi:10.1371/journal.pgen.1003874 24763277 10.1371/journal.pgen.1003874 PMC3998918

[26] Attardo GM, Benoit JB, Michalkova V, Patrick KR, Krause TB, et al (2014) The Homeodomain protein Ladybird Late Regulates Synthesis of Milk Proteins during Pregnancy in the Tsetse Fly (*Glossina morsitans*). PLoS Negl Trop Dis 10: e2645 doi:10.1371/journal.pntd.0002645 10.1371/journal.pntd.0002645 PMC3998940 24763082

[27] Benoit JB, Hansen IA, Attardo GM, Michalková V, Mireji PO, et al (2014) Aquaporins are critical for provision of water during lactation and progeny hydration to maintain tsetse fly reproductive success. PLoS Negl Trop Dis 8: e2517 doi:10.1371/journal.pntd.0002517 24762803 10.1371/journal.pntd.0002517 PMC3998938

[28] Michalkova V, Benoit JB, Attardo GM, Medlock J, Aksoy S (2014) Prevention of oxidative stress during insect viviparous reproduction is critical to prolonged reproductive output and longevity. PLoS ONE 9: e87554 doi:10.1371/journal.pone.0087554 24763119 10.1371/journal.pone.0087554 PMC3998933

[29] Alam U, Medlock J, Brelsfoard C, Pais R, Lohs C, et al (2011) *Wolbachia* symbiont infections induce strong cytoplasmic incompatibility in the tsetse fly *Glossina morsitans* . PLoS Pathog 7: e1002415. 22174680 10.1371/journal.ppat.1002415 PMC3234226

[30] Brelsfoard C, Tsiamis G, Falchetto M, Gomulski L, Telleria E, et al (2014) Presence of Extensive *Wolbachia* Symbiont Insertions Discovered in the Genome of Its Host *Glossina morsitans morsitans* . PLoS Negl Trop Dis 8: e2728 doi:10.1371/journal.pntd.0002728 24763283 10.1371/journal.pntd.0002728 PMC3998919

[31] Rose C, Belmonte R, Armstrong S, Molyneux G, Haines L, et al (2014) An investigation into the protein composition of the *Glossina morsitans morsitans* peritrophic matrix. PLoS Negl Trop Dis 8: e2691 doi:10.1371/journal.pntd.0002691 24763256 10.1371/journal.pntd.0002691 PMC3998921

[32] Telleria EL, Benoit JB, Zhao X, Savage AF, Regmi S, et al (2014) Insights into the trypanosome transmission process revealed through transcriptomic analysis of parasitized tsetse salivary glands. PLoS Negl Trop Dis 8: e2649 doi:10.1371/journal.pntd.0002649 24763140 10.1371/journal.pntd.0002649 PMC3998935

